# LIPI-4 as a Critical Modulator of InlB-Mediated Pathogenicity in *Listeria monocytogenes*

**DOI:** 10.3390/microorganisms14030645

**Published:** 2026-03-12

**Authors:** Yatao Qi, Wenjuan Zhao, Caixia Liu, Ruixuan Qian, Lu Liu, Zhongke Yin, Xun Ma, Jing Wang

**Affiliations:** 1 College of Animal Science and Technology, Shihezi University, Shihezi 832000, China; 18094801903@163.com (Y.Q.); liucaixia0402@163.com (C.L.); 15373481428@163.com (R.Q.); liulu0626666@163.com (L.L.); yzk1849608975@163.com (Z.Y.); 2 Institute of Animal Husbandry and Veterinary Science, Xinjiang Academy of Agricultural Reclamation Sciences, Shihezi 832000, China; zwj-130@163.com; 3 National Key Laboratory of Genetic Improvement and Healthy Sheep Breeding, Shihezi 832000, China

**Keywords:** *Listeria monocytogenes*, *inlB*, LIPI-4, virulence, bacterial pathogenesis

## Abstract

*Listeria monocytogenes* (Lm) is a foodborne pathogen whose virulence depends on the coordinated action of multiple virulence factors. Although deletion of either LIPI-4 or *inlB* reduces the virulence of Listeria monocytogenes, it remains unknown whether these two factors are functionally or regulatory connected. Therefore, we constructed an *inlB* deletion mutant and its complemented strain in the Lm928 and *ΔLIPI-4* backgrounds. We assessed bacterial growth, biofilm formation, motility, host cell interactions (adhesion, invasion, intracellular proliferation), plaque formation, mouse organ colonization. Growth curve analysis showed no significant differences among strains. qPCR revealed that LIPI-4 modulates *inlB* expression in a cell-type-specific manner: *inlB* was downregulated in *ΔLIPI-4* under culture and HTR-8 infection, but upregulated during hCMEC/D3 infection—yet functional defects persisted in all cases. Biofilm assays showed that *ΔLIPI-4* and the double mutant exhibited enhanced biofilm formation, with the double mutant exceeding *ΔLIPI-4*, demonstrating synergistic enhancement. Motility assays indicated that LIPI-4 dominates bacterial movement, with *ΔLIPI-4* and the double mutant showing identical severe defects. Plaque formation analysis showed that LIPI-4 is essential for cell-to-cell spread, while *inlB* deletion unexpectedly enhanced plaque formation—an effect completely abolished in the absence of LIPI-4. Host cell assays across Caco-2, HTR-8, and hCMEC/D3 models revealed that LIPI-4 is the core determinant of adhesion, invasion, and intracellular proliferation, whereas *inlB* contributes in the context of LIPI-4 and its effects vary with the specific cellular process examined. In mice, LIPI-4 was essential for systemic colonization of the liver and spleen, with *inlB* acting as a co-factor, whereas *inlB* unexpectedly promoted higher bacterial burdens in the brain, suggesting that *inlB* modulates LIPI-4-mediated neuroinvasion. Overall, our results establish LIPI-4 as the central determinant of Lm virulence, with *inlB* acting as a context-dependent co-factor that modulates LIPI-4-mediated pathogenesis in a cell type- and tissue-specific manner.

## 1. Introduction

*Listeria monocytogenes* (Lm) is a Gram-positive zoonotic bacterium and the principal etiologic agent of listeriosis [1]. Most mild cases are limited to gastrointestinal symptoms, but in susceptible populations, including newborns, pregnant women, and the elderly, Lm can cause severe invasive outcomes such as meningitis and sepsis [2,3,4,5,6,7]. In pregnant women, it is particularly responsible for miscarriage, stillbirth, or premature birth, with a case fatality rate exceeding 20% [8,9]. The pathogen exhibits remarkable environmental resilience, capable of growth across a wide range of temperatures (1–45 °C), pH (4.5–9), and NaCl concentrations up to 20% [10,11,12,13]. This combination of pathogenicity and environmental hardiness places Lm among the four major foodborne pathogens.

A key aspect of Lm is its ability to invade host cells and disseminate across intestinal, blood-brain, and placental barriers [3,14,15]. To achieve this, Lm deploys multiple virulence factors to invade non-phagocytic cells, among which InlA (internalin A) and InlB (internalin B) are recognized as key invasins [16]. InlA specifically binds to host E-cadherin via its N-terminal leucine-rich repeat domain, primarily mediating bacterial adhesion [17]. Notably, natural mutations introducing a premature stop codon in the *inlA* gene result in truncation of InlA. Such mutations are more frequently observed in food and environmental isolates but are relatively rare in clinical isolates, suggesting an association with attenuated virulence. However, some strains carrying truncated InlA have still been reported to cause clinical infections.

In contrast to InlA, InlB exhibits a broader host cell tropism. It associates with the bacterial surface through its C-terminal GW repeat domains via non-covalent interactions with lipoteichoic acid, and recognizes a diverse set of receptors, including c-Met (hepatocyte growth factor receptor (HGFR)) and gC1q-R (C1q-binding protein), and glycosaminoglycans [18,19]. Although InlB is generally considered a key invasin of *Listeria*, its functional contribution during infection appears to be strain-dependent and critically influenced by its expression level. Phelps, C.C. et al. [20] demonstrated that in the *prfA* wild-type strain 10403S, InlB was sufficient to activate downstream signaling via c-Met yet incapable of independently mediating bacterial internalization. Only upon introduction of the prfA* mutation, which markedly elevates InlB expression, did its invasive function become apparent. Consistent with this, Lamond, N.M. et al. [21] reported that the cardiac tissue-adherent strain 07PF0776 displayed significantly higher surface-bound InlB levels than strain 10403S. This elevated expression enabled InlB to play a critical role in vertical transmission from the placenta to the fetus, and knockout of *inlB* completely abolished this advantage. In conclusion, InlB not only acts as an invader to facilitate the entry of bacteria into host cells, but also regulates the tissue affinity and pathogenic potential of Listeria through its unique domain organization and expression level.

To date, multiple pathogenicity islands have been identified in Lm, mainly including *Listeria* pathogenicity island-1 (LIPI-1), LIPI-3, and LIPI-4. LIPI-1 contains six major genes (*prfA*, *plcA*, *hly*, *mpl*, *actA*, *plcB*) and is primarily involved in the bacterium’s intracellular parasitism and direct cell-to-cell spread, serving as the core gene cluster for the intracellular life cycle of Lm [16]. LIPI-3 comprises eight genes (*llsA*, *llsG*, *llsH*, *llsX*, *llsB*, *llsY*, *llsD*, *llsP*), which encode Listeriolysin S (LLS) [22]. This toxin aids the bacterium’s survival in the gastrointestinal tract and helps modulate the host’s intestinal microbiota in the early stages of infection to promote colonization. Another important virulence determinant, the *Listeria* pathogenicity island 4 (LIPI-4), was first reported by Maury et al. [23] and is implicated in maternal–fetal and neurological listeriosis. LIPI-4 encodes a maltose-6′-phosphate glucosidase, a transcription antitermination factor, an uncharacterized PTS system-related protein, and the EIIA, EIIB, and EIIC components [24]. Experimental evidence has independently established the respective roles of *inlB* and LIPI-4 in Lm virulence. Deletion of *inlB* has been shown to impair adhesion, invasion, proliferation [25], and overall virulence [26]. This virulence defect parallels that seen with mutations in LIPI-4; deletion of its EIIC component [27] or the entire cluster [28] reduces pathogenicity. Moreover, LIPI-4 deletion specifically compromises adhesion to diverse host cell lines (hCMEC/D3, HTR-8, RAW264.7) [29]. Given the overlapping phenotypic outcomes of *inlB* and LIPI-4 disruption, it remains unclear whether these two virulence factors interact functionally or exhibit synergistic regulation.

To address this question, this study systematically compares parental and mutant strains by examining core phenotypes, including adhesion, invasion, intracellular proliferation, biofilm formation, motility, and pathogenicity in mice. This approach aims to clarify the functional relationship between LIPI-4 and *inlB*, providing new insights into Lm tissue tropism and its mechanisms for crossing host barriers.

## 2. Materials and Methods

### 2.1. Materials

#### 2.1.1. Microbial Strains, Plasmids, and Cell Cultures

The strain Lm928 was isolated and characterized from frozen chicken by the Food Testing Center of Xinjiang Academy of Agriculture and Reclamation Sciences. It is currently maintained in the culture collection of the College of Animal Science and Technology, Shihezi University. The LIPI-4 deletion strain (*ΔLIPI-4*) was successfully generated by Tingyu Ruan. Escherichia coli DH5ɑ was purchased from TransGen Biotech (Beijing, China). The plasmid pMD19-T (Simple), a TA cloning vector used for the ligation of PCR products with A-tailing, was acquired from TaKaRa (Baobio, Dalian, China). The temperature-sensitive shuttle plasmid pKSV7 was kindly provided by Professor Weihuan Fang of the Laboratory of Molecular Microbiology and Food Safety, School of Animal Sciences, Zhejiang University. The integration plasmid pIMK2, containing the strong constitutive promoter pHelp, was a gift from Associate Professor Yin Yue-lan of Yangzhou University, and is also stored in our laboratory.

The immortalized human cerebral microvascular endothelial cell line hCMEC/D3 (BNCC337728, BeNa Culture Collection, Xinyang, China) was cultured in DMEM (Gibco, Grand Island, NY, USA) supplemented with 10% fetal bovine serum (FBS) (Excell Bio, Shanghai, China). The human chorionic trophoblast cell line HTR-8/Svneo (BNCC359649, BeNa Culture Collection) was maintained in RPMI-1640 medium (Gibco, Suzhou, China) containing 10% FBS. The human colon adenocarcinoma cell line Caco-2 (CL-0050, Procell, Wuhan, China) was grown in DMEM with 20% FBS. The murine fibroblast cell line NCTC clone 929 (L929, CL-0137, Procell) was cultured in MEM (Procell, Wuhan, China) with 10% FBS. All cell lines were incubated at 37 °C in a humidified atmosphere of 5% CO_2_.

#### 2.1.2. Key Reagents

The brain heart infusion (BHI) medium was from Qingdao Hi-Tech Park Haibo Biotechnology Co., Ltd. (Qingdao, China).; the agarose gel electrophoresis recovery kit, 2× Taq Master Mix (Dye Plus), DL2000 DNA Marker, 5K DNA Marker, and whole-genome DNA extraction kits were products of Nanjing Nuoweizan Biotechnology Co., Ltd. (Nanjing China); Pfu enzyme and dNTPs came from Beijing Quantype Gold Biotechnology Co., Ltd. (Beijing China); ampicillin (Amp), HEPES, chloramphenicol, lysozyme, and kanamycin were obtained from Beijing Boaotuoda Technology Co., Ltd. (Beijing China); T4 DNA ligase was acquired from Promega (Beijing, China); and restriction endonucleases *BamH*I, *Pst*I, and *Xho*I were sourced from TaKaRa.

#### 2.1.3. Animals

Six- to eight-week-old Kunming mice were obtained from the Animal Experimental Center of Xinjiang Medical University and housed under controlled conditions (21–26 °C, 40–70% relative humidity). All procedures were approved by the Biology Ethics Committee of Shihezi University (Approval A2021-26) and conducted in accordance with the Chinese National Guidelines for Housing and Care of Laboratory Animals.

### 2.2. Methods

#### 2.2.1. Construction of the *inlB* Gene Deletion Mutant and Complemented Strain

Based on the complete gene sequence of LM09-00558 from GenBank (accession number GCA_001565535.2), specific primers were designed with Oligo 6.24 software (Table 1).

The experimental procedures followed the methodology of Li H. [30]. The genomic DNA of the Lm strain Lm928 was extracted using a bacterial genomic DNA extraction kit. Using the Lm928 genome as a template, the upstream and downstream homologous arms of the *inlB* gene were amplified by PCR and subsequently purified. These purified homologous arms were then used as templates for splice overlap extension PCR (SOE-PCR) to generate a fusion fragment, which was also purified.

The fusion fragment was ligated into the pMD19-T vector using T4 DNA ligase in an overnight reaction at 4 °C. The ligation product was transformed into *E. coli* DH5α competent cells via the heat-shock method. The resulting recombinant plasmid, pMD19-T-*ΔinlB*, was verified by double restriction enzyme digestion and DNA sequencing.

The verified pMD19-T-*ΔinlB* plasmid and the pKSV7 plasmid were separately digested with *BamH*I and *Pst*I. The target fragments were purified and then co-transformed into DH5α competent cells using the heat-shock method. The constructed product was confirmed by double enzyme digestion.

Competent cells were prepared from the wild-type Lm928 strain and the *ΔLIPI-4* mutant strain. Single colonies of Lm928 and *ΔLIPI-4* were inoculated into BHI broth and cultured overnight at 37 °C with shaking at 160 rpm. One milliliter of the overnight culture was transferred into 50 mL of sucrose-supplemented BHI broth (supplemented with 0.342 g of sucrose per 100 mL of BHI broth) and grown under the same conditions until the OD_600_ reached approximately 0.2 (about 4.5–5 h). Penicillin G was then added to the culture at a final concentration of 0.015 mg/mL (15 μL of a 50 mg/mL stock solution added to 50 mL culture), and incubation continued for an additional 2 h. Cells were harvested by centrifugation at 8000 rpm for 10 min at 4 °C, washed twice with ice-cold HEPES buffer (1 mM HEPES, 0.5 M sucrose, pH 7.0), using 50 mL for the first wash and 30 mL for the second wash. The cells were then resuspended in 5 mL of the same buffer. Lysozyme was added to the resuspension buffer at a final concentration of 0.15 mg/mL (15 μL of a 50 mg/mL stock solution added to 5 mL of buffer), and the suspension was mixed thoroughly and incubated in a 37 °C water bath for 20 min. After another centrifugation step (8000 rpm, 10 min, 4 °C), the pellet was washed twice with HEPES buffer (30 mL per wash), centrifuged again under the same conditions, and finally resuspended in 1 mL of HEPES buffer. The competent cells were aliquoted (100 μL per tube) and stored at −80 °C until use. The verified recombinant pKSV7 plasmid was introduced into these competent cells via electroporation (2.5 kV, 5 ms). Homologous recombination was induced at 42 °C under chloramphenicol selection (10 μg/mL). Subsequently, the cultures were passaged at 37 °C in the absence of antibiotics to facilitate plasmid loss. The strains that had lost the plasmid were further passaged 10 times at 37 °C to ensure stable inheritance of the mutation. The resulting strains were designated *ΔinlB* and *ΔLIPI-4-ΔinlB*, respectively.

To construct the complemented *ΔinlB* strain (*ΔinlB*::*inlB*), we employed the previously reported strong constitutive promoter pHelp to ensure robust gene expression. The full-length *inlB* gene was amplified by PCR using Lm928 genomic DNA as the template and primers *inlB*-internal-F and *inlB*-internal-R. The amplified gene was cloned into the pMD19-T vector, and the construct was verified by double enzyme digestion and sequencing. The verified plasmid and the pIMK2 vector were then digested with *Xho*I and *Pst*I, respectively. The digested *inlB* fragment was ligated into the linearized pIMK2 vector. The ligation product was transformed into DH5α via heat-shock, and the correct construct was confirmed by double enzyme digestion before being electroporated into *ΔinlB* competent cells. The transformed strain was passaged 10 times at 37 °C with shaking at 160 rpm, with PCR verification performed every five passages. The final complemented strain was designated *ΔinlB*::*inlB*.

#### 2.2.2. Recover Bacteria

Prior to experiments, the five frozen bacterial strains—Lm928, *ΔinlB*, *ΔLIPI-4*, *ΔLIPI-4-ΔinlB*, and the complemented strain *ΔinlB*::*inlB*—were recovered from frozen stocks stored at −80 °C. They were streaked onto BHI agar plates using the four-quadrant method and incubated at 37 °C for 20 h.

#### 2.2.3. Real-Time PCR

Bacterial RNA Extraction and qRT-PCR Analysis.

Single colonies of bacterial strains were inoculated into BHI broth and cultured overnight at 37 °C with shaking at 160 rpm. Bacterial pellets were collected, ground in liquid nitrogen, and total RNA was extracted using an RNA extraction kit (TransGen Biotech, Beijing, China) according to the manufacturer’s instructions. RNA was reverse-transcribed into cDNA using a reverse transcription kit (Takara Bio, Dalian, China), this cDNA was then used for the qRT-PCR reaction using PerfectStart^®^ Green qPCR SuperMix (TransGen Biotech, Beijing, China) according to the manufacturer’s protocol on the LightCycler 96 (Roche, Basel, Switzerland).

Bacterial RNA Extraction from Infected Host Cells.

Host cells were seeded in 60 mm dishes containing 4 mL of culture medium. At the time of infection, hCMEC/D3 cells were at approximately 80% confluence (~3.6 × 10^6^ cells/dish) and HTR-8 cells at ~5 × 10^6^ cells/dish. Overnight bacterial cultures were added at a multiplicity of infection (MOI) of 10. After 1 h of infection, the medium was replaced with fresh medium containing 100 μg/mL gentamicin (to kill extracellular bacteria). One hour later, the medium was again replaced with a medium containing 10 μg/mL gentamicin (to inhibit residual extracellular growth during prolonged incubation). At 13 h post-infection, cells were harvested by trypsinization, and total RNA was extracted following the same procedure described above. qRT-PCR was subsequently performed.

Primer sequences used for qRT-PCR are listed in Table S1.

#### 2.2.4. Determination of the Bacterial Growth Curve

The growth curve was determined as described by Weidi Shi et al. [31]. A single colony of each strain was inoculated into 2 mL of BHI broth and grown at 37 °C for 12 h with shaking (160 rpm), followed by adjustment of the culture density to a standardized OD_600_. To determine the growth curve, 200 μL of bacterial suspension was used to inoculate 20 mL of BHI broth, followed by incubation at 37 °C with shaking at 160 rpm for 12 h. The OD_600_ was measured and recorded at 2 h intervals.

#### 2.2.5. Bacterial Motility

Bacterial motility was also assessed according to the method of Weidi Shi et al. [31]. A 100 mL volume of semi-solid medium was prepared, consisting of 2% NaCl, 1% tryptone, and 0.25% agar. Single colonies were inoculated into 2 mL of BHI broth and grown at 37 °C for 12 h with shaking (160 rpm), followed by adjustment of the culture density to a consistent OD_600_, and 1 μL of each culture was spot-inoculated onto the semi-solid agar. Plates were incubated statically at 28 °C for 24 and 48 h. Images were captured, and the diameters of the migration halos were measured.

#### 2.2.6. Biofilm Formation

Biofilm formation was assessed as described previously [32]. Single colonies were inoculated into BHI broth and cultured at 37 °C with shaking (160 rpm) for 12 h. The bacterial cultures were then adjusted to an OD_600_ of 0.2 and seeded into a 48-well cell culture plate. The plates were then incubated statically at 37 °C to allow biofilm formation. Following incubation, biofilms formed at 24, 48, and 72 h were stained with 1% crystal violet, destained with 95% ethanol, and the dissolved dye was quantified by measuring OD_600_.

#### 2.2.7. Plaque Assay

Plaque formation was determined as described previously [33]. L929 cell monolayers in 6-well plates (at a density of approximately 2.2 × 10^6^ cells per well) were infected at a multiplicity of infection (MOI) of 0.1. After 1 h of incubation at 37 °C under 5% CO_2_, the inoculum was removed, and the cells were gently washed with phosphate-buffered saline (PBS) to eliminate non-adherent bacteria. The monolayers were then incubated for 1 h with fresh medium containing 100 μg/mL gentamicin to kill any remaining extracellular bacteria. Subsequently, the cells were overlaid with 1.4% low-melting-point agarose in medium supplemented with 10 μg/mL gentamicin to restrict bacterial movement to cell-to-cell spread. Following 24 h incubation, the monolayers were fixed and stained with crystal violet for plaque visualization and counting.

#### 2.2.8. Adhesion, Invasion, and Intracellular Proliferation Assays

Established methods [34] were adhered to. For adhesion, invasion, and intracellular proliferation assays, Caco-2, HTR-8, and hCMEC/D3 cells were seeded in 12-well plates and cultured overnight at 37 °C under 5% CO_2_ until reaching approximately 80% confluence (corresponding to approximately 5.6 × 10^5^, 4.0 × 10^5^, and 3.2 × 10^5^ cells/well, respectively). The medium was then replaced with antibiotic-free medium. Bacterial strains from overnight cultures were washed with PBS and diluted to a MOI of 10 in the same antibiotic-free medium. The bacterial suspension was added to the cells and incubated for 1 h.

To quantify adhered bacteria, the cells were lysed with 0.2% Triton X-100 at 1 h post-infection, and the lysates were serially diluted and plated on BHI agar (this time point was defined as 0 h for the proliferation assay). Immediately afterward, fresh medium containing 100 μg/mL gentamicin was added to kill extracellular bacteria. After an additional 1 h of incubation (2 h total post-infection), cells were lysed to enumerate intracellular (invaded) bacteria.

For the intracellular proliferation assay, the medium was replaced with medium containing 10 μg/mL gentamicin following the invasion time point. Cell lysates were collected at 4, 8, and 12 h after the initial infection (i.e., 3, 7, and 11 h after adding the low-concentration gentamicin). All lysates were serially diluted, plated on BHI agar, and incubated at 37 °C for 20–22 h before colony counting.

#### 2.2.9. Bacterial Load in Mice

Bacterial strains were prepared as described in Section 2.2.4. The bacterial suspensions were adjusted to a uniform OD_600_ and diluted two-fold to achieve a target inoculum of approximately 1 × 10^7^ CFU in a 100 μL volume for intraperitoneal injection. A total of 43 Kunming mice were randomly allocated into seven groups: a blank control group (PBS; *n* = 3), and groups infected with the Lm928, *ΔinlB*, *ΔLIPI-4*, *ΔLIPI-4-ΔinlB*, and the complemented strain *ΔinlB*::*inlB* (*n* = 8 per group). At 48 h post-infection, the mice were euthanized. The liver, spleen, and brain were aseptically collected, homogenized, and subjected to serially diluted and plated on BHI agar for bacterial enumeration (CFU counting).

#### 2.2.10. Statistical Analysis

Data are shown as mean ± SEM. Statistical analysis was conducted with SPSS 20.0 software. All datasets met the assumption of homogeneity of variances (assessed by Levene’s test, *p* > 0.05). For comparisons among multiple groups, one-way ANOVA with Tukey’s honest significant difference (HSD) post hoc test was applied. A *p*-value below 0.05 was defined as statistically significant.

## 3. Results

### 3.1. LIPI-4 Positively Regulates inlB Expression

**Figure 1:**
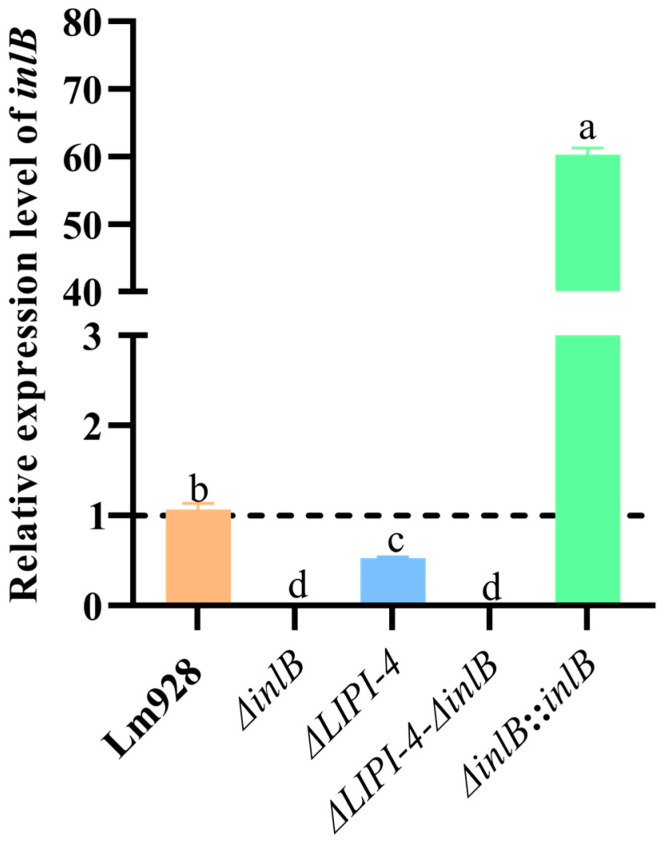
Bacterial strains were cultured overnight (14–16 h) at 37 °C with shaking at 160 rpm, washed with PBS, and homogenized in liquid nitrogen. Total RNA was extracted using a commercial RNA extraction kit and subsequently reverse-transcribed into cDNA using a reverse transcription kit for qPCR analysis. Values are presented as mean ± SEM of three biological independent replicates. Different letters indicate significant differences (*p* < 0.05, Tukey’s HSD test). Expression levels of target genes in Lm strains are shown relative to the wild-type control (dashed line, value = 1).

To investigate the functional relationship between *inlB* and LIPI-4, we constructed in-frame deletion mutants (*ΔinlB* and *ΔLIPI-4-ΔinlB*) and a chromosomal complementation strain (*ΔinlB*::*inlB*). Successful construction of all strains was confirmed by PCR (Figures S1 and S2). The five strains were cultured at 37 °C with shaking at 160 rpm, and total RNA was extracted for qPCR analysis (Figure 1). Compared with the wild-type Lm928, the *inlB* transcripts in *ΔinlB* and *ΔLIPI-4-ΔinlB* were undetectable, indicating successful knockout of the *inlB* gene. It is worth noting that the *inlB* mRNA level of *ΔLIPI-4* decreased to 0.53-fold of that in Lm928 (*p* < 0.05). In contrast, the *inlB* mRNA level in the complemented strain *ΔinlB*::*inlB* was significantly higher than that in Lm928 (*p* < 0.05).

### 3.2. Deletion of inlB and/or LIPI-4 Does Not Affect Bacterial Growth

**Figure 2:**
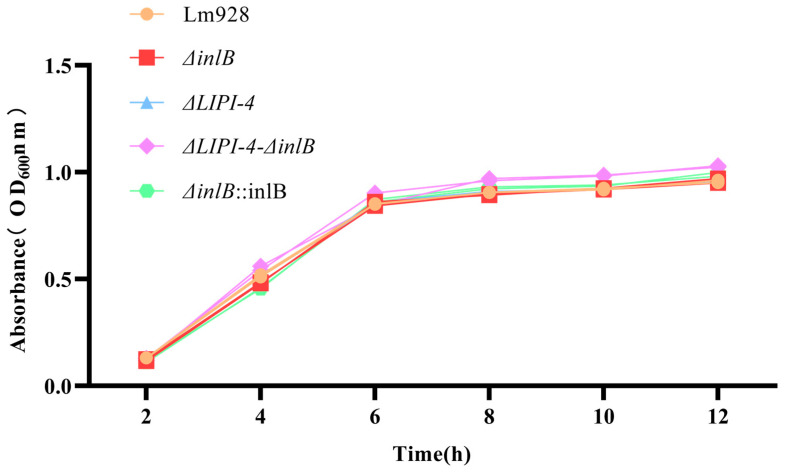
Growth curves of Lm strains in BHI medium at 37 °C. Values are presented as mean ± SEM of three biological independent replicates.

The growth kinetics of the five strains were monitored under standard conditions (37 °C, 160 rpm). No statistically significant differences in optical density (OD_600_) were observed among the strains at any of the six time points measured (*p* > 0.05) (Figure 2).

### 3.3. Combined Deletion of inlB and LIPI-4 Synergistically Enhances Biofilm Formation

**Figure 3:**
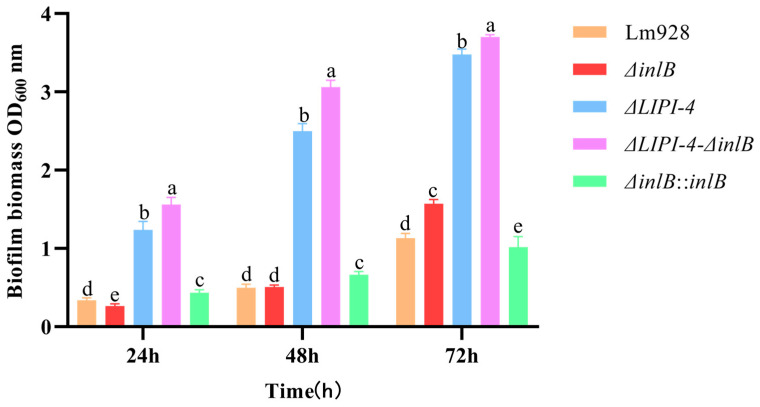
Effects of LIPI-4 and *inlB* on the biofilm formation ability of Lm. The five strains were adjusted to the same optical density, inoculated into a 48-well plate, and incubated statically at 37 °C for 24, 48, and 72 h. Biofilm formation was then quantified by crystal violet staining followed by OD measurement. Values are presented as mean ± SEM of three biological independent replicates. Different letters indicate significant differences (*p* < 0.05, Tukey’s HSD test).

Biofilm formation was quantified under static conditions at 37 °C (Figure 3). At 24 h, both the double-mutant strain and *ΔLIPI-4* exhibited significantly enhanced biofilm formation compared to Lm928 (*p* < 0.05), with the double mutant showing the most pronounced increase (*p* < 0.05). In contrast, *ΔinlB* displayed no significant enhancement relative to the parental strain. At 48 h, the biofilm-forming capacities of the double mutant and *ΔLIPI-4* remained significantly greater than that of Lm928 (*p* < 0.05), with the double mutant strain outperforming *ΔLIPI-4* (*p* < 0.05). No significant difference was observed between *ΔinlB* and Lm928 at this time point (*p* > 0.05). By 72 h, both the double mutant and *ΔLIPI-4* continued to show stronger biofilm formation than Lm928 (*p* < 0.05); notably, *ΔinlB* also exhibited a significant increase compared to Lm928 at this late stage (*p* < 0.05). Furthermore, this enhanced phenotype was successfully restored in the corresponding complementation strain.

### 3.4. LIPI-4 Is Essential for Bacterial Motility, While inlB Plays a Modulatory Role

**Figure 4:**
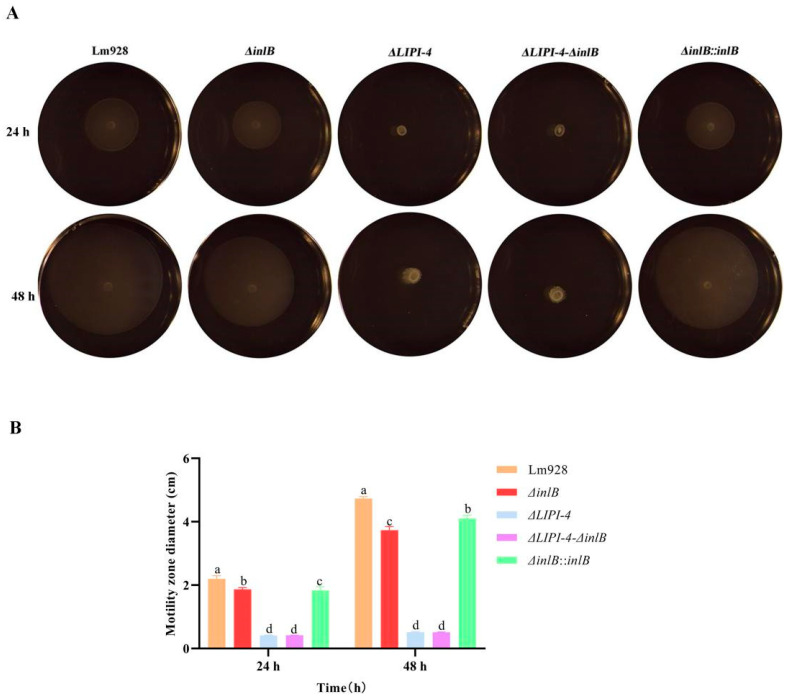
Effects of LIPI-4 and *inlB* on the motility of Lm. The optical densities of the five strains were first adjusted to the same value. Subsequently, 1 μL of each bacterial suspension was spot-inoculated onto TSA plates. The plates were then incubated statically at 28 °C for 24 h and 48 h. (**A**) Representative images of the motility halos. (**B**) Quantitative measurement of the halo diameters. Values are presented as mean ± SEM of three biological independent replicates. Different letters indicate significant differences (*p* < 0.05, Tukey’s HSD test).

Bacterial motility was assessed on semi-solid medium at 28 °C (Figure 4). After 24 h and 48 h of incubation, similar motility patterns were observed. *ΔLIPI-4* and the double mutant *ΔLIPI-4-ΔinlB* exhibited the smallest migration zones, with no significant difference between them (*p* > 0.05). *ΔinlB* and its complemented strain *ΔinlB*::*inlB* showed intermediate motility, both significantly lower than Lm928 (*p* < 0.05); although a statistical difference was detected between these two strains, they behaved similarly.

### 3.5. ΔinlB Increases Plaque Formation, an Effect Abolished by LIPI-4 Deletion

**Figure 5:**
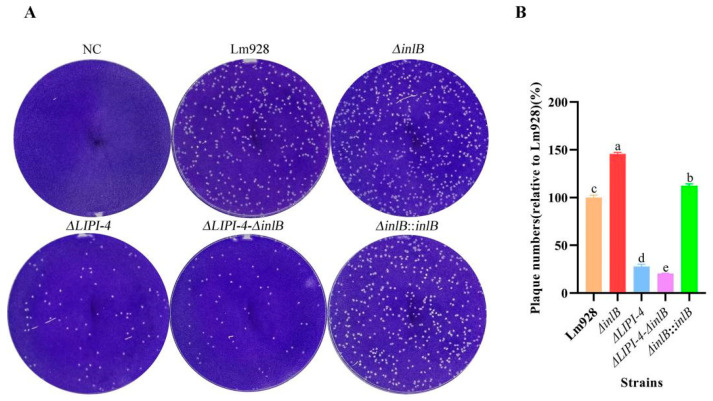
LIPI-4 is essential for efficient cell-to-cell spread of Lm in plaque assays. (**A**) Representative images of crystal violet-stained L929 cell monolayers showing clear lytic plaques after 24 h infection. NC (negative control, uninfected cells). (**B**) The extent of bacterial spread is presented as the relative plaque number (%) compared to the wild-type strain Lm928. Values are presented as mean ± SEM of three biological independent replicates. Different letters indicate significant differences (*p* < 0.05, Tukey’s HSD test).

We assessed cell-to-cell spread using a plaque assay in L929 cell monolayers (Figure 5A,B). The results revealed that both *ΔinlB* and its complemented strain *ΔinlB*::*inlB* formed significantly more plaques than the wild type strain (*p* < 0.05). In contrast, *ΔLIPI-4* and the double mutant *ΔLIPI-4-ΔinlB* exhibited significantly fewer plaques than the wild-type (*p* < 0.05).

### 3.6. LIPI-4 Is Required for InlB-Mediated Invasion and Intracellular Proliferation in a Cell Type-Dependent Manner

To assess the pathogenicity of different Lm strains, we evaluated bacterial adhesion, invasion, and intracellular proliferation using Caco-2, HTR-8, and hCMEC/D3 cell models.

**Figure 6:**
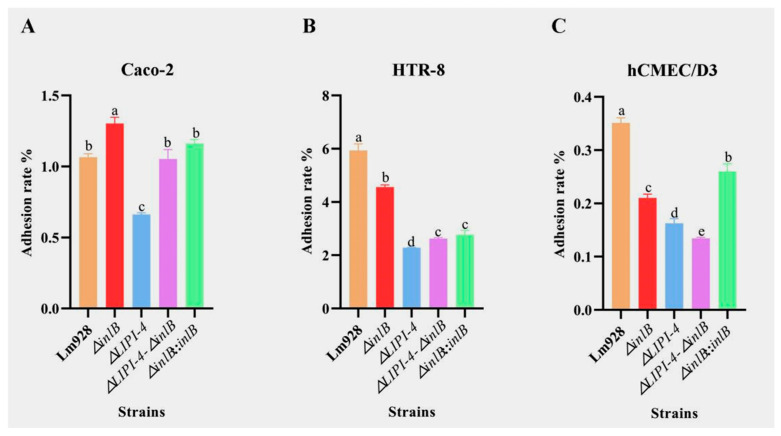
Adhesion of Lm in Caco-2 (**A**), HTR-8 (**B**), and hCMEC/D3 cells (**C**). Adhesion efficiency was determined 1 h post-infection. Values are presented as mean ± SEM of three biological independent replicates. Different letters indicate significant differences (*p* < 0.05, Tukey’s HSD test).

Adhesion to different host cell lines (Figure 6)

The adhesion patterns of Lm strains varied markedly across the three cell types (Figure 6A–C). In Caco-2 cells (Figure 6A), *ΔinlB* unexpectedly showed significantly higher adhesion than wild-type Lm928 (*p* < 0.05), whereas *ΔLIPI-4* showed a marked reduction. Both the *ΔLIPI-4-ΔinlB* double mutant and the complemented strain *ΔinlB*::*inlB* restored adhesion to Lm928 levels (*p* > 0.05).

In contrast, in HTR-8 cells (Figure 6B), *ΔinlB* showed significantly lower adhesion than Lm928 (*p* < 0.05), as did all other mutant strains. *ΔinlB* exhibited significantly higher adhesion than *ΔLIPI-4* (*p* < 0.05). The double mutant and the complemented strain did not differ significantly from each other (*p* > 0.05), and both were intermediate between *ΔinlB* and *ΔLIPI-4*.

In hCMEC/D3 cells (Figure 6C), *ΔinlB* also showed significantly lower adhesion than Lm928, but remained significantly higher than *ΔLIPI-4* (*p* < 0.05). The double mutant exhibited even lower adhesion than *ΔLIPI-4* alone (*p* < 0.05).

**Figure 7:**
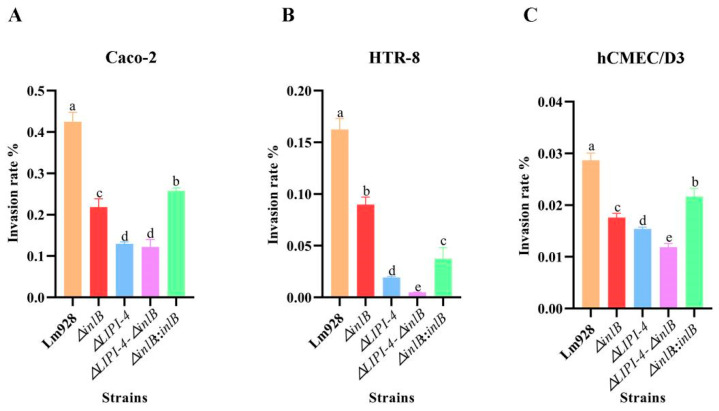
Invasion of Lm in Caco-2 (**A**), HTR-8 (**B**), and hCMEC/D3 cells (**C**). Invasion efficiency was assessed after 2 h of infection (including 1 h of gentamicin treatment to kill extracellular bacteria). Values are presented as mean ± SEM of three biological independent replicates. Different letters indicate significant differences (*p* < 0.05, Tukey’s HSD test).

Invasion efficiency across host cell lines (Figure 7)

All mutant strains showed significantly reduced invasion compared to wild-type strain in all three cell lines, but the magnitude of reduction varied (Figure 7A–C).

In Caco-2 cells (Figure 7A), *ΔLIPI-4* and the double mutant exhibited the lowest invasion rates, both significantly below that of *ΔinlB* (*p* < 0.05). *ΔinlB* and its complemented strain both showed reduced invasion compared to wild-type, with the complemented strain only partially restoring invasion ability.

In HTR-8 cells (Figure 7B), *ΔinlB* was significantly more invasive than *ΔLIPI-4* (*p* < 0.05), and both were significantly more invasive than the double mutant (*p* < 0.05). These results indicate that both *inlB* and LIPI-4 contribute to host cell invasion, with LIPI-4 playing a more dominant role in this cell type.

In hCMEC/D3 cells (Figure 7C), *ΔinlB* was less invasive than Lm928 but more invasive than *ΔLIPI-4* (*p* < 0.05), and the double mutant showed significantly reduced invasion compared to *ΔLIPI-4* (*p* < 0.05).

**Figure 8:**
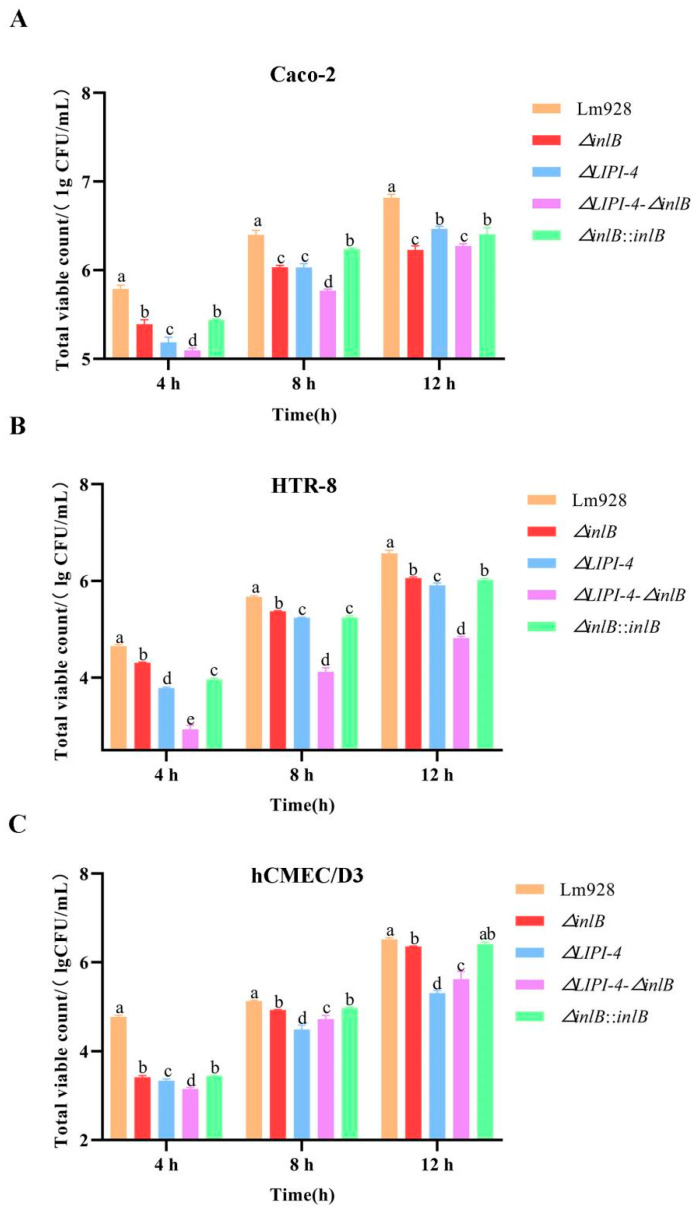
Intracellular proliferation of Lm in Caco-2 (**A**), HTR-8 (**B**), and hCMEC/D3 cells (**C**). Intracellular proliferation was monitored at 4, 8, and 12 h after the initial gentamicin treatment. Values are presented as mean ± SEM of three biological independent replicates. Different letters indicate significant differences (*p* < 0.05, Tukey’s HSD test).

Intracellular proliferation across host cell lines (Figure 8)

Intracellular proliferation was assessed at 4, 8, and 12 h post-infection in all three cell lines (Figure 8A–C). All mutant strains showed significantly lower bacterial counts than Lm928 at all time points across all cell lines (*p* < 0.05), with the exception of the time-dependent shifts noted below.

In Caco-2 cells (Figure 8A), the double mutant consistently showed the lowest proliferation levels. *ΔLIPI-4* exhibited a time-dependent shift: it was more impaired at early time points but became the least impaired mutant by 12 h, second only to wild-type. *ΔinlB* and its complemented strain showed similar proliferation patterns throughout the time course, both remaining significantly below wild-type.

In HTR-8 cells (Figure 8B), *ΔinlB* showed the highest counts among mutants, followed by *ΔLIPI-4*, while the double mutant consistently had the lowest. The complemented strain was significantly lower than *ΔinlB* at 4 and 8 h (*p* < 0.05), but showed no significant difference at 12 h (*p* > 0.05).

In hCMEC/D3 cells (Figure 8C), *ΔinlB* consistently showed higher counts than *ΔLIPI-4* and the double mutant (*p* < 0.05), while no significant difference was detected between *ΔinlB* and its complemented strain (*p* > 0.05). Notably, at 4 h, *ΔLIPI-4* counts were significantly higher than those of the double mutant (*p* < 0.05); however, this trend reversed at 8 h and 12 h, with the double mutant surpassing *ΔLIPI-4*.

To investigate whether the cell-type-specific proliferation phenotypes correlate with differential gene expression, we examined *inlB* transcript levels in *ΔLIPI-4*-infected cells at 12 h post-infection (Figure S3). In HTR-8 cells, where *ΔLIPI-4* showed sustained proliferation defects, *inlB* expression was significantly downregulated (*p* < 0.01). In contrast, in hCMEC/D3 cells, where *ΔLIPI-4* was eventually outpaced by the double mutant, *inlB* expression was significantly upregulated (*p* < 0.05).

### 3.7. LIPI-4 Is the Core Virulence Factor for Systemic Dissemination, While inlB Plays a Tissue-Specific Role in Brain Infection

**Figure 9:**
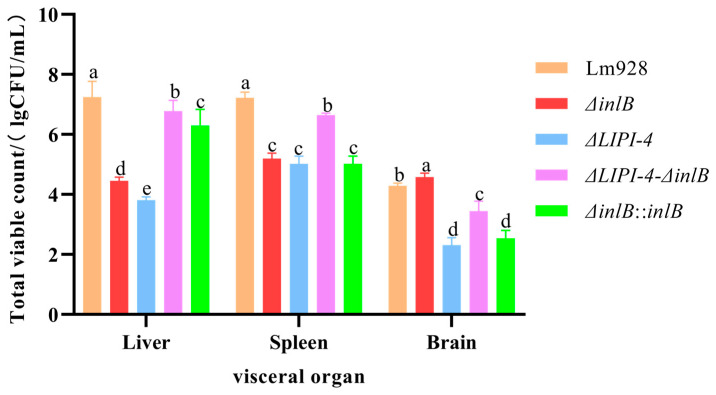
Assessment of Lm virulence in mice: bacterial burden in organs. On day 0 (the day of challenge), mice (*n* = 43 total; *n* = 8 per experimental group and *n* = 3 for PBS control) were monitored for body weight daily and humanely euthanized on day 2 post-infection. Bacterial loads in the liver, spleen, and brain of infected mice were quantified. Bacterial enumeration from organs was performed by homogenizing the brain, liver, and spleen in Triton X-100 using a mechanical homogenizer. The homogenates underwent serial dilution, were spread-plated on BHI agar, and incubated at 37 °C. Colonies were counted after incubation. Different letters indicate significant differences (*p* < 0.05, Tukey’s HSD test).

To definitively assess the roles of LIPI-4 and *inlB* in pathogenesis, mice were challenged with the wild-type and mutant strains, and bacterial burdens in the liver, spleen, and brain were quantified (Figure 9). In the liver and spleen, the wild-type strain Lm928 achieved the highest bacterial loads, establishing the benchmark for systemic infection. The hierarchy observed (Lm928 > *ΔLIPI-4-ΔinlB* > *ΔinlB*) (*p* < 0.05) demonstrates that *inlB* acts as a co-factor to support LIPI-4-mediated colonization of these organs. Strikingly, a tissue-specific role for *inlB* was uncovered in the brain. Here, the *ΔinlB* mutant exhibited the highest bacterial burden, significantly exceeding that of Lm928 (*p* < 0.05).

## 4. Discussion

Lm is a foodborne pathogen whose virulence depends on the coordinated expression of multiple virulence factors. This study examined the interaction between *inlB* and LIPI-4, revealing a hierarchical and synergistic regulatory relationship between the two.

In assays of fundamental physiological traits, no significant differences were observed in the growth curves among the various knockout and complemented strains compared to the wild-type strain. This indicates that the absence of *inlB* and LIPI-4 does not impair bacterial growth capacity, effectively ruling out growth-related effects on other phenotypic traits. Building on this finding, we further demonstrated that LIPI-4 plays an indispensable role in environmental adaptation. In motility assays, although *inlB* also significantly influenced the motility zone size, the effect of LIPI-4 was more pronounced. Notably, the motility zone of the double mutant did not differ significantly from that of the LIPI-4 single knockout, suggesting a functional interaction between *inlB* and LIPI-4 in the regulation of motility. Liu C. et al. [29] demonstrated that LIPI-4 deficiency significantly impairs bacterial motility. They further showed that LIPI-4 promotes flagellar assembly via metabolic regulation, which in turn influences motility. The study by Jiang X. et al. [35] showed that double mutation of the *virAB* and *virS* genes downregulates *flaA* expression. In related research, Guo Q. et al. [36] reported that deletion of the *virS*/*virR* genes in Lm 10403S significantly impaired bacterial motility, and further found that this deletion also significantly downregulated transcription of the chemotaxis gene *cheY*. These results collectively suggest that loss of LIPI-4 may similarly lead to downregulation of the chemotaxis system function. Moreover, given the functional interplay between InlB and LIPI-4 observed in this study, such a mechanism provides a plausible explanation for the observed phenotype. Indeed, at 72 h, biofilm formation by the *ΔinlB* strain was significantly increased, whereas complementation of *inlB* resulted in a marked reduction in biofilm formation. A comparative analysis of the *ΔLIPI-4* and the double-deficient strains at this time point revealed that the expression of both LIPI-4 and *inlB* is unfavorable for biofilm formation. Moreover, the concurrent deletion of these two genes led to a further significant enhancement of biofilm formation, suggesting a potential synergistic effect in the regulation of this process. Franciosa G. et al. [37] have shown that the biofilm-forming ability of strains expressing truncated InlA was significantly higher than that of strains expressing full-length InlA. This phenomenon of reduced virulence accompanied by enhanced biofilm formation is similar to the findings of the present study, indicating that virulence is weakened by the loss of LIPI-4 and *inlB*, which may in turn promote biofilm formation. In addition, a study by Suo Y. et al. [38] showed that an ABC transporter (encoded by the gene *Im.G_1771*) inhibits *Listeria* biofilm formation by negatively regulating the expression of superoxide dismutase (SOD), since the antioxidant effect of SOD is essential for scavenging reactive oxygen species and promoting biofilm formation. Therefore, it is possible that LIPI-4 and *inlB* also contribute to biofilm formation by positively regulating SOD. Taken together, it remains to be verified whether the effects of LIPI-4 and *inlB* on biofilm-forming ability result from their hypovirulent phenotype or through indirect regulation of other biofilm-related genes.

Plaque assay results identified LIPI-4 as a core factor required for the establishment of efficient initial bacterial infection. Notably, deletion of *inlB* enhanced bacterial intracellular survival and intercellular transmission in L929 cells. As reported by Chatterjee A. et al. [39], ethanolamine is a degradation product of both bacterial and eukaryotic membrane phospholipids. In Lm, the regulatory gene *eutV* and metabolic gene *eutB* significantly enhance intracellular survival and replication. We hypothesize that LIPI-4 upregulates the *eut* operon, thereby driving efficient ethanolamine utilization to promote robust bacterial cell survival.

In adhesion assays using Caco-2 cells, the *ΔinlB* mutant exhibited an increased adhesion rate. However, the adhesion rates of both the double mutant (*ΔLIPI-4-ΔinlB*) and the complemented strain (*ΔinlB*::*inlB*) were significantly lower than that of *ΔinlB*, indicating that this enhancement in adhesion is associated with LIPI-4. It has been reported that ActA-mediated bacterial aggregation is critical for enhancing adhesion efficiency and invasion probability, a process driven by the master regulator PrfA [40]. In addition, subsequent invasion assays further underscored the essential role of LIPI-4. The reduced invasion of the *inlB* mutant likely stems from the failure of large bacterial aggregates—formed during adhesion—to be efficiently internalized by host cells. Consequently, these aggregates remain trapped on the cell surface, ultimately leading to a significantly lower number of internalized bacteria compared to the wild-type strain. According to Fang X. et al. [41], Lm GalE facilitates the galactosylation of wall teichoic acid (WTA), thereby creating a stable anchoring platform for InlB on the bacterial surface. In the absence of GalE, this platform fails to assemble; consequently, even when InlB is abundantly synthesized, it cannot be properly displayed on the cell surface to mediate infection, leading to significant attenuation of virulence. In the present study, adhesion and invasion assays using HTR-8 cells demonstrated that LIPI-4 deficiency severely compromises both bacterial adhesion and invasion. Given the functional association between InlB and LIPI-4, we propose that the proteins encoded by LIPI-4 may directly or indirectly modify the bacterial cell surface. This modification is hypothesized to create a requisite structural environment for the stable anchorage and proper conformational display of the InlB protein.

Intracellular proliferation of Lm requires successful escape from phagosomes and entry into the nutrient-rich cytoplasm. Recent studies have shown that following internalization, the invasion protein InlB recruits the host type III PI3K Vps34 and its product, PI3P, thereby accelerating the conversion of bacteria-containing vesicles into Rab7-positive late endosomes. This process optimizes LLO-mediated phagosomal escape and enhances bacterial proliferation in cells such as HeLa [42]. In contrast, our data from Caco-2, HTR-8, and hCMEC/D3 cells consistently and strongly demonstrate that InlB’s ability to promote intracellular proliferation is closely linked to the presence of LIPI-4. Notably, in Caco-2 cells, this association exhibits a clear temporal pattern: the absence of *inlB* has a relatively limited impact on proliferation early in infection (4 h), whereas its essential role becomes fully evident at mid-to-late stages (8–12 h). This contrasts with the “early-acceleration” effect reported in the HeLa model. We propose that *inlB* acquires its full functional capacity only through this “licensing” by LIPI-4, enabling it to efficiently perform downstream functions such as Vps34 recruitment.

Our in vivo mouse experiments yielded results consistent with the in vitro cell culture findings. In hCMEC/D3 cells, the absence of either *inlB* or LIPI-4 significantly reduced bacterial adhesion and invasion, with the double-mutant strain exhibiting an even greater reduction. This indicates a synergistic effect of LIPI-4 and *inlB* on bacterial adhesion and invasion in these cells and underscores the dominant role of LIPI-4 in this process. An interesting shift was observed during intracellular proliferation: while the trend at 4 h post-infection mirrored the initial adhesion/invasion phenotype, the bacterial count of the *ΔLIPI-4-ΔinlB* double mutant exceeded that of the *ΔLIPI-4* single mutant by 8 h. Furthermore, although the *ΔinlB* mutant count remained lower than that of the wild-type Lm928 at 12 h, the difference between them continued to narrow. In the mouse infection model, brain bacterial loads revealed that both the *ΔLIPI-4* and *ΔLIPI-4-ΔinlB* strains were significantly lower than Lm928, confirming that deletion of LIPI-4 alone is sufficient to abolish high-level brain colonization and that its core function is independent of *inlB*. Intriguingly, the bacterial load of the double mutant was higher than that of the *ΔLIPI-4* single mutant. This subtle phenotype suggests that in the absence of the primary LIPI-4 virulence mechanism, the concurrent loss of *inlB* may subtly alter bacterial physiology or permit the engagement of low-efficiency alternative invasion pathways—a possibility warranting future investigation. Second, the deletion of *inlB* alone significantly increased the brain bacterial burden. This aligns with the trend observed in the hCMEC/D3 cell proliferation assay, where the defect of the *ΔinlB* mutant relative to the wild type diminished over time. This convergence indicates that the impaired initial invasion—a hallmark of *inlB* deficiency—may confer a long-term advantage within the specific niche of the brain. This observation is consistent with the report by Werbrouck H. et al. [43], which suggested that reduced invasion ability (often linked to low *inlB* expression in clinical isolates) may attenuate pro-inflammatory innate immune responses (e.g., IL-8), thereby promoting immune evasion and cryptic colonization. Jung C. et al. [44] identified CD44v6 as an essential co-receptor for c-Met during InlB-mediated invasion of endothelial cells, where it acts as a critical signal amplifier for downstream pathway activation. Their work demonstrates that InlB binding to c-Met depends on CD44v6, with the three components forming a complex that promotes bacterial entry. In parallel, Eriksson E. et al. [45] showed that CD44 supports the intracellular survival and replication of Lm within macrophages and fibroblasts. Integrating these insights, we propose the following testable hypothesis: In the presence of *inlB*, bacteria efficiently invade endothelial cells via the InlB–c-Met–CD44v6 axis. In the absence of *inlB*, bacterial invasion shifts toward a macrophage-dependent route that relies on CD44-facilitated intracellular survival—a pathway proven to enhance bacterial replication. This shift could allow bacteria that reach the brain via the macrophage-mediated “Trojan horse” route to establish robust replication foci more effectively, ultimately leading to a higher cerebral bacterial load.

In our study, the complemented strain was constructed using the site-specific integrative plasmid pIMK2, which carries the strong constitutive promoter Phelp. The recombinant plasmid was integrated into the chromosome of the deletion mutant, enabling stable and constitutive expression of the target gene [46]. qPCR confirmed that this construct successfully and significantly upregulated *inlB* expression. However, in subsequent functional assays, complementation failed to rescue the *ΔinlB* phenotype in some tests, with results even mirroring those of the *ΔinlB* mutant. Similar phenomena have been reported; for instance, one study noted that overexpression of the *yjbH* gene in a complemented strain yielded biological characteristics indistinguishable from the deletion mutant [47]. Furthermore, research has suggested that the precise recapitulation of the native state is challenging due to alterations in the genetic context and topological structure of the complemented gene [48]. Therefore, our results may also be attributed to such factors. However, the results of adhesion, invasion and intracellular proliferation of HTR-8 cells showed that the complemented strain performed weaker than *ΔinlB*. Interestingly, we found that *inlB* expression was downregulated in the *ΔLIPI-4* strain specifically in HTR-8 cells, while the complemented strain itself overexpresses *inlB* due to the strong constitutive promoter. Together, these observations suggest that in HTR-8, *inlB* may require precise regulation of expression to function properly.

Although our data suggest a functional interplay between LIPI-4 and *inlB*, we cannot exclude the possibility that *inlB* may act independently. A definitive demonstration of functional dependency would therefore require overexpression of *inlB* in the *ΔLIPI-4* background. Beyond this, we acknowledge that the broader mechanistic basis of this relationship remains to be fully elucidated. The current study lacks direct molecular evidence, such as comparative expression analyses, assessment of *inlB* surface localization, or receptor-binding assays. These mechanistic aspects represent a crucial avenue for our future investigations.

In summary, this study systematically demonstrates, through both in vivo and in vitro experiments, a previously unrecognized hierarchical synergy between LIPI-4 and *inlB* in the pathogenicity of Lm. Specifically, LIPI-4 acts as a key virulence determinant that provides the essential molecular foundation for the function of downstream effectors such as *inlB*. In contrast, *inlB* serves as a dynamic fine-tuning element whose activity is closely linked to LIPI-4, with both factors forming an integrated virulence module. The complex, and at times contradictory, phenotypes of *inlB* across different infection stages underscore the sophistication and context-dependency of this regulatory network. Our findings not only confirm the central role of LIPI-4 but also reveal how this module enables the bacterium to flexibly adjust its invasion, colonization, and immune evasion strategies in response to diverse host microenvironments. This work, by elucidating an integrated virulence module governed by hierarchical synergy between LIPI-4 and *inlB*, provides a new conceptual framework for understanding Lm pathogenesis from a systems-level perspective.

## Figures and Tables

**Table 1:** Primers used in this study.

Primers	Sequence (5′—3′)	Target Fragment/Purpose	Restriction Site	Product Length (bp)
*inlB*-up-F	**GG**GGTACCTAACGGAGGGAACACTACACC	It was used for amplification of the upstream homology arm of the *inlB* gene.	*BamH*I	324
*inlB*-up-R	AAATAGCTTTTCGTAGGACTATCCTCTCCTTGAT			
*inlB*-down-F	ATCAAGGAGAGGATAGTCCTACGAAAAGCTATTT	It was used for amplification of the downstream homology arm of the *inlB* gene.		409
*inlB*-down-R	**CG**GGATCCCGATTCTTGCTAGACCACCAG		*Pst*I	
*inlB*-flank-F	TAATGACGGTGTAACAACATC	It was used for amplification of the regions flanking *inlB* for verification of gene deletion.		3437 (1544)
*inlB*-flank-R	AATAATTTAATGCGTAGCCTC			
*inlB*-internal-F	**AA**CTGCAGGTGAAAGAAAAGCACAACC	It was used for amplification of the *inlB* gene.	*Xho*I	1893
*inlB*-internal-R	**CC**CTCGAGTTTAAGGGCACAGAAATGA		*Pst*I	

The genomic DNA of the protective bases are indicated in bold, and restriction sites are underlined.

## Data Availability

The original contributions presented in the study are included in the article, further inquiries can be directed to the corresponding authors.

## Supplementary Materials

The following supporting information can be downloaded at https://www.mdpi.com/article/10.3390/microorganisms14030645/s1, Table S1: The information of Real-time PCR primers in this study; Figure S1: PCR detection during the process of constructing the missing strain; Figure S2: PCR detection during the process of constructing the compensation stock; Figure S3: The qPCR results of *inlB* regulation by LIPI-4 in different host cells.

## Abbreviations

The following abbreviations are used in this manuscript.

Lm	*Listeria monocytogenes*
InlB	internalin B
c-Met	Hepatocyte Growth Factor Receptor
LIPI-4	*Listeria* pathogenicity island 4
FBS	fetal bovine serum
BHI	Brain heart infusion
Amp	ampicillin
SOE-PCR	splice overlap extension PCR
*E. coli*	*Escherichia coli*
PBS	Phosphate-buffered saline
MOI	multiplicity of infection
CFU	Colony-forming unit
HEPES	4-(2-Hydroxyethyl)-1-piperazineethanesulfonic acid
NC	negative control
