# Supplementary material for: LIPI-4 as a Critical Modulator of InlB-Mediated Pathogenicity in Listeria monocytogenes
Source: Microorganisms. 2026 Mar 12;14(3):645. doi: 10.3390/microorganisms14030645 (PMC13028617; doi:10.3390/microorganisms14030645)
Supplement: Supplementary file 1 [file microorganisms-14-00645-s001.zip › microorganisms-4135051-supplementary.pdf]

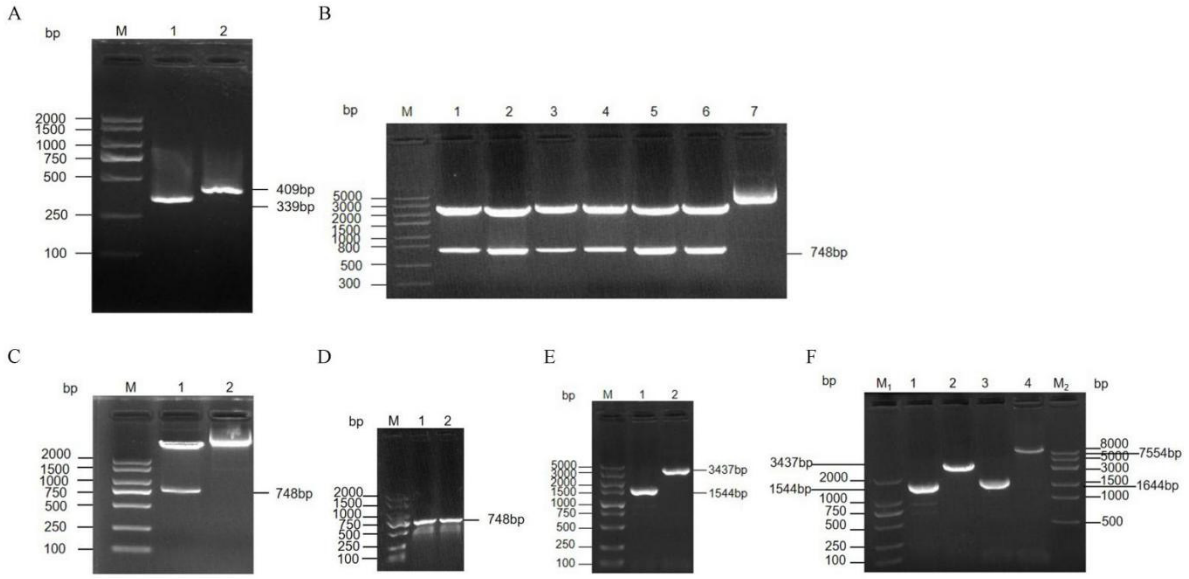

Figure S1. Verification of the construction of deletion mutants by PCR and restriction digestion. (A) Amplification of upstream and downstream homologous arms of *inlB*. M: DL2000 DNA marker; 1: upstream homologous arm (339 bp); 2: downstream homologous arm (409 bp). (B) Double restriction enzyme digestion verification of pMD19-T after fragment ligation and integration. M: Trans 5K DNA marker; 1-6: pMD19-T after double restriction enzyme digestion; 7: undigested plasmid. (C) Double restriction enzyme digestion verification of the fusion fragment ligation. M: Trans 8K DNA marker; 1: pKSV7 after double restriction enzyme digestion; 2: undigested pKSV7. (D) Screening of electroporants. M: DL2000 DNA marker; 1: positive transformant of Lm928; 2: positive transformant of *ΔLIPI-4*. (E) Validation of *ΔinlB*. M: Trans 5K DNA marker; 1: *ΔinlB* (1544 bp); 2: Lm928 (3437 bp). (F) Validation of the *ΔLIPI-4-ΔinlB* double mutant. M1: Trans 2K DNA marker; M2: Trans 8K DNA marker; 1: *ΔLIPI-4-ΔinlB* strain; 2, 4: Lm928 (wild-type control). 3: Verification of LIPI-4 deletion.

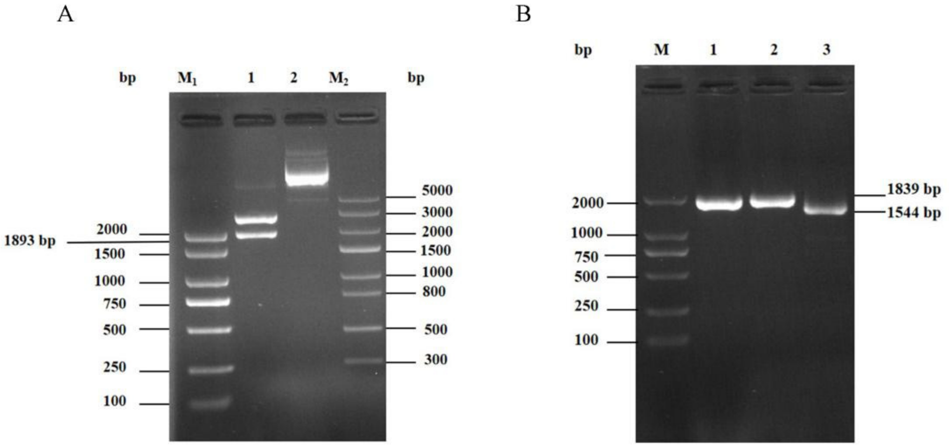

Figure S2. Construction and validation of the *inlB* complemented strain. (A) Verification of pIMK2 by double restriction enzyme digestion. M1: DL2000 DNA marker; M2: Trans 5K DNA marker; 1: pIMK2 after double restriction enzyme digestion; 2: undigested pIMK2. (B) Validation of the *ΔinlB::inlB* complemented strain. M: Trans 2K DNA marker; 1: *ΔinlB::inlB* strain amplified with complementation primers; 2: Lm928 wild-type strain amplified with complementation primers; 3: *ΔinlB::inlB* strain amplified with flanking primers.

Table S1. Target gene primer sequences used for RT-PCR.

Gene Name	Forward primer sequence (5′→3′)	Reverse primer sequence (5′→3′)	Amplicon size (bp)
gyrB	AGACGCTATTGATGCCGATGA	GTATTGCGCGTTGTCTTCGA	91
inlB	GTGAAAGAAAAGCACAACCCAAG	TCGCCCCGTTTCCAATAATTAT	94

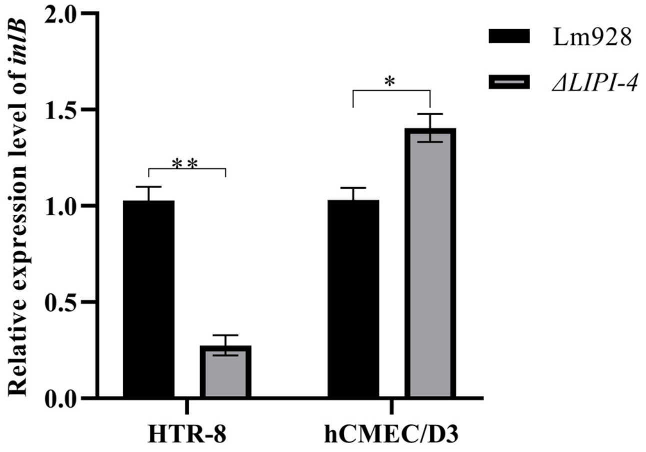

Figure S3. HTR-8 and hCMEC/D3 cells were seeded in 60 mm cell culture dishes. Upon reaching approximately 80% confluence, the cells were infected with *Listeria monocytogenes* at a multiplicity of infection (MOI) of 10. After 1 hour of infection, the medium was replaced with fresh medium containing 100 μg/mL gentamicin. One hour later, the medium was again replaced with medium containing 10 μg/mL gentamicin. At 13 hours post-infection, cells were harvested by trypsinization, and total RNA was extracted using liquid nitrogen grinding followed by qPCR analysis. Data are presented as mean ± SEM from three independent experiments.* $p < 0.05$, ** $p < 0.01$.
